# Changes in Alcohol Consumption During and After the Covid-19 Pandemic From 2020 to 2023 in a Prospective Cohort of Italian Adults

**DOI:** 10.2188/jea.JE20230340

**Published:** 2025-02-05

**Authors:** Sonia Cerrai, Giulia Carreras, Filippo Monti, Chiara Stival, Alessandra Lugo, Cristina Bosetti, Silvia Biagioni, Tiziana Fanucchi, Giuseppe Gorini, Andrea Amerio, Luisa Mastrobattista, Claudia Mortali, Anna Odone, Sabrina Molinaro, Luc Smits, Silvano Gallus, the “Lost in Italy” and “Lost in Toscana” Study Investigators

**Affiliations:** 1 National Research Council, Institute of Clinical Physiology (CNR-IFC), Pisa, Italy; 2 Department of Epidemiology, Care and Public Health Research Institute (CAPHRI), Maastricht University, Maastricht, The Netherlands; 3 Institute for Cancer Research, Prevention and Clinical Network (ISPRO), Florence, Italy; 4 Department of Medical Epidemiology, Istituto di Ricerche Farmacologiche Mario Negri IRCCS, Milan, Italy; 5 Department of Surgical, Medical and Molecular Pathology and Critical Care Medicine, University of Pisa, Pisa, Italy; 6 Unit of Health Promotion, Epidemiology, and Government of Territorial Demand, ASST Fatebenefratelli Sacco, Milan, Italy; 7 University of Genoa, Genoa, Italy; 8 Istituto Superiore di Sanità, Rome, Italy; 9 Department of Public Health, Experimental and Forensic Medicine, University of Pavia, Pavia, Italy

**Keywords:** alcohol, at-risk drinking, AUDIT-C, COVID-19, prospective cohort

## Abstract

**Background:**

The lockdowns imposed by the government during coronavirus disease 2019 (COVID-19) pandemic have had a significant impact on the Italian population habits.

**Methods:**

LOckdown and lifeSTyles in Italy and in Tuscany studies collected data on a representative sample of the Italian adult population in 2020 (*n* = 6,003) followed up through 2023 via four additional surveys (3,000 ≤ *n* ≤ 6,600) through an online self-administered questionnaire. The Alcohol Use Disorders Identification Test-Concise was used to identify at-risk drinkers. Considering the cohort of individuals who took part to the first and at least one other wave (*n* = 5,378), a multilevel logistic model was used to derive odds ratios (ORs) and corresponding 95% confidence intervals (CIs) of being at-risk drinkers.

**Results:**

The prevalence of at-risk drinkers was 26.4% before, 23.4% during the first lockdown, and stabilized around 30.0% thereafter. Being at-risk alcohol consumers decreased with decreasing economic status (*P* for trend <0.001), was less frequent among middle-aged compared to younger (OR 0.73; 95% CI, 0.60–0.89) and among divorced/separated (OR 0.77; 95% CI, 0.60–0.99) or single (OR 0.75; 95% CI, 0.64–0.89) compared to married individuals. It was more frequent among individuals with anxiety or depressive symptoms (OR 1.24; 95% CI, 1.12–1.37), those using psychotropic drugs (OR 1.99; 95% CI, 1.69–2.35) and users of conventional and/or alternative nicotine products (OR 3.67; 95% CI, 3.00–4.48).

**Conclusion:**

The long-term trends in alcohol consumption after the COVID-19 pandemic are unfavorable in Italy. The results point to an increased vulnerability for at-risk alcohol consumption among younger individuals, women with higher economic status, and married individuals. At-risk drinking is strongly related to mental health symptoms and nicotine consumption.

## INTRODUCTION

The coronavirus disease 2019 (COVID-19) pandemic had a vast impact on many facets of people’s daily lives worldwide.^1^^–^^3^ Like many other countries, Italy has experienced several waves of the COVID-19 pandemic, each characterized by government-imposed restrictions and significant changes in daily life.^4^^,^^5^ Besides the direct health consequences—such as established increase in body weight,^6^ sleep disorders,^7^ anxiety and depressive symptoms,^8^^,^^9^ and sexual dysfunctions^10^^,^^11^—containment measures and periods of lockdown have led to significant changes in social behaviors.^9^^,^^12^^,^^13^ Among these, changes in substance use in general, and alcohol use in particular, emerged as a particularly interesting aspect, as they are closely linked to social interaction and may potentially influence people’s wellbeing.^14^

During the COVID-19 pandemic, alcohol use has likely been influenced by multiple factors, including stress, social isolation, anxiety, and restrictions on travel and social activities,^15^^–^^17^ while the availability of alcohol has never waned. Despite the relevance of this topic, there is currently a lack of comprehensive understanding regarding alcohol consumption dynamics during the various phases of the pandemic in Italy, particularly in the long term. Although increases in alcohol consumption and risky drinking behaviors have been observed in many European countries, including Italy,^18^^,^^19^ previous studies have primarily focused on specific subgroups or lacked representative samples.^20^^–^^25^ Therefore, it is important to further investigate how alcohol consumption has changed during and after different pandemic waves.

The present study addresses this gap in knowledge by examining variations in alcohol use and at-risk alcohol consumption in the Italian adult population between 2020 and 2023, using representative samples within the study LOckdown and lifeSTyle IN ITALY (‘Lost in Italy’)^26^ and its extension ‘Lost in Toscana’.^27^ The large majority of the study population was followed up. Taking advantage of the cohort of individuals prospectively followed in subsequent waves, the study also aims to identify potential subgroups of the population who may be more vulnerable to the negative effects of alcohol use during emergency periods.

## METHODS

### Study design and population

Doxa, the Italian branch of the Worldwide Independent Network/Gallup International Association, conducted the investigation. The LOST in Italy study consists of a survey (Survey 1) on a sample of 6,003 participants aged 18–74 years. It is representative of the Italian population regarding various demographic characteristics (age, sex, socio-economic characteristics, and geographic area)—extracted from a Doxa online panel, who completed a web-based interview during the first and strictest Italian COVID-19 lockdown, between April 27 and May 3, 2020. This survey collected data regarding two different time points: the lockdown period (March 9–May 3, 2020; t_0_) and the pre-lockdown period (February, 2020; t_−1_). Regarding this last point, the questionnaire specifies that it pertains “to pre-emergency “normality” (early February, 2020)”, in order to have an estimation of the general habits before the onset of the pandemic and restrictions. Following surveys, mostly based on the same study participants of Survey 1, were carried out in correspondence with the main COVID-19 waves (eFigure 1). A second and a third longitudinal surveys (Survey 2 and 3) were conducted on about half of the initial cohort (*n* = 3,185) in November 27–December 20, 2020 (t_1_), and (*n* = 3,000) in May 7–18, 2021 (t_2_). Two years after the beginning of the COVID-19 pandemic, between February 24 and March 21, 2022, 6,600 participants took part to a comparable web-based survey (Survey 4) within the Lost in Toscana study (t_3_), a nationally representative survey with an over-sample of the Tuscany region population. Lastly, a follow-up of the LOST in Toscana study (Survey 5) was conducted between April 4 and May 3, 2023 (t_4_) on 6,600 participants. In each follow-up survey, some individuals were lost; therefore, to maintain the sample’s representativeness, new individuals were added from the appropriate strata in each survey. For the present study, individuals who took part in the first survey and in at least one of the following surveys were selected (*n* = 5,378).

### Ethical aspects

The two study protocols of the Lost in Italy and Lost in Tuscany studies were approved by the Ethics Committee of the coordinating centers (Istituto Besta, file number: 71-73, April 2020, and Comitato Etico Regionale per la Sperimentazione Clinica della Toscana, Sezione Area Vasta Centro, file number: CEAVC 19834, April 2021, respectively). All the enrolled participants provided informed consent to participate in the study.

### Outcome variable

Alcohol drinkers were defined as those individuals who did not report “none” to the question “How often do you actually consume an alcoholic beverage?”. The Alcohol Use Disorders Identification Test - Concise (AUDIT-C) was used to identify at-risk alcohol drinkers,^28^ meaning individuals who exceed moderate drinking limits and may be at risk of physical or social harm related to alcohol. The AUDIT-C consists of three questions investigating: (i) monthly frequency of alcoholic beverages consumption, (ii) number of alcoholic beverages that are consumed on a typical drinking day, considering one alcoholic unit equal to 12 g of alcohol (ie, one can of beer [330 mL], one glass of wine [125 mL], one aperitif [80 mL] or one spirit [40 mL]), and (iii) frequency of binge drinking, defined as the intake of six or more alcoholic beverages on a single occasion. To each answer a score from 0 to 4 is assigned; the total AUDIT-C score ranges from 0 to 12, with a score ≥4 for women and ≥5 for men indicating at-risk alcohol drinking.

### Independent variables

Using self-administered questionnaires, information was collected on socio-demographic characteristics (age, sex, level of education, geographic area of residence, economic status, working condition, and marital status) and lifestyle habits and addictive behaviors, including conventional cigarette smoking and use of novel nicotine-based products. Non-users were considered subjects who used neither conventional cigarettes nor novel products, while dual users were considered individuals who reported using at least two products among conventional tobacco, e-cigarettes, or heated tobacco products (HTPs).

Mental health variables were also considered (anxiety or depressive symptoms, quantity and quality of sleep, and quality of life), as well as consumption of selected psychotropic drugs, namely-antidepressants, anxiolytics/benzodiazepines, hypnotics, antipsychotics, and mood stabilizers.^26^^,^^29^ Individuals scoring higher than 3 in the 2-item generalized anxiety disorder (GAD-2) scale^30^ or in the 2-item Patient Health Questionnaire (PHQ-2) scale^31^ were identified as presenting anxiety or depressive symptoms. Individuals who reported sleeping less than 7 hours per night or who rated their overall sleep as fairly poor or very poor were identified as presenting sleep disorders.^32^ Individuals reporting the use of at least one drug among antidepressants, hypnotics, anxiolytics/benzodiazepine, antipsychotics and mood stabilizers were considered psychotropic drug users.

### Statistical analysis

*Repeated representative surveys:* descriptive analyses were carried out to provide prevalence of current alcohol use and at-risk alcohol drinking in the representative samples of Italian adults included in the Lost in Italy and Lost in Tuscany studies, at each different time point. In order to guarantee the representativeness of each sample, a statistical weight was applied in each survey.

*Prospective cohort analysis:* being at-risk alcohol drinker was then investigated in association with survey time and with socio-demographic variables, psychological distress symptoms, and use of conventional cigarettes and/or novel products within the cohort of individuals who participated to the first and at least one other survey. A multilevel random intercept logistic model adjusted by geographic area of residence was used to take into account the dependency of observations within subjects, also allowing the inclusion of individuals who participated in different number of surveys.^27^ In this model, some of the socio-demographic variables, such as sex, geographic area of residence, education, and age, were fixed at baseline, whereas marital status, economic status, and working condition were time-varying. Variables on mental distress, such as anxiety or depressive symptoms, sleep disorders, use of psychotropic drugs, and use of conventional cigarette and/or novel products, were also assumed to be time-varying. All statistical analyses were performed using Stata Version 17 (StataCorp, College Station, TX, USA).

## RESULTS

### Prevalence of alcohol consumption and at-risk drinking during the COVID-19 pandemic

**Figure 1.**
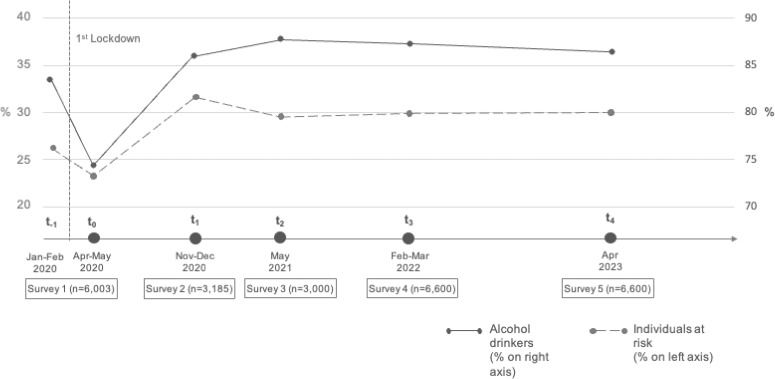
Prevalence of alcohol consumers and at-risk alcohol drinkers at each survey

The prevalence of individuals according to current alcohol consumption and at-risk alcohol consumption in all the surveys is presented in Figure 1 and stratified by sex and survey time in Table 1. Unlike in Carreras 2022, in Table 1 and eTable 1 we presented unweighted counts in order to better highlight the sample dimension and distribution. The prevalence of alcohol drinkers decreased from 83.5% (95% confidence interval [CI], 82.3–84.7%) in the pre-lockdown period (t_−1_) to 74.4% (95% CI, 73.0–75.8%) during the lockdown period (t_0_). Then, it increased again, remaining higher in all the subsequent surveys compared to the pre-lockdown period, ranging from 86.0% to 87.8%. A similar trend was observed for mean AUDIT-C score: mean score was 2.9 (standard deviation [SD], 2.3) at t_−1_, it slightly decreased to 2.6 (SD, 2.5) during the lockdown period (t_0_), and it increased, reaching 3.2 (SD, 2.3–2.5), in the subsequent surveys. The prevalence of at-risk alcohol drinkers slightly decreased from 26.4% (95% CI, 25.0–27.9%) at t_−1_ to 23.4% (95% CI, 22.1–24.8%) at t_0_, but increased again reaching 31.7% (95% CI, 29.6–31.2%) at t_1_, 29.7% (95% CI, 27.6–31.8%) at t_2_, 29.9% (95% CI, 28.5–31.3%) at t_3_ and 30.2% (95% CI, 28.8–31.6%) in the last survey (t_4_).

**Table 1.** Distribution of individuals according to alcohol consumption and at-risk consumption, by sex and survey (raw data and weighted prevalence), Italy, 2020–2023

	Survey 1 t_−1_^a^	Survey 1 t_0_^a^	Survey 2 t_1_^a^	Survey 3 t_2_^a^	Survey 4 t_3_^a^	Survey 5 t_4_^a^
Total sample, *N*	6,003	6,003	3,185	3,000	6,600	6,600
Men, *N*	3,026^*^	3,026	1,689	1,601	3,299	3,288
Women, *N*	2,977^*^	2,977	1,496	1,399	3,301	3,312
Alcohol drinkers, % (95% CI)						
Total sample	83.5 (82.3–84.7)	74.4 (73.0–75.8)	86.0 (83.9–87.3)	87.8 (86.1–89.2)	87.3 (86.2–88.3)	86.5 (85.4–87.6)
Men	89.9 (88.4–91.3)	82.0 (80.0–83.8)	92.5 (90.4–94.2)	91.7 (89.6–93.4)	92.7 (91.5–93.6)	92.0 (90.6–93.2)
Women	77.3 (75.3–79.1)	67.0 (64.8–69.1)	79.0 (76.2–81.6)	83.9 (81.3–86.2)	82.0 (80.2–86.3)	81.0 (79.2–82.7)
Score AUDIT-C, mean (SD)						
Total sample	2.9 (2.3)	2.6 (2.5)	3.2 (2.5)	3.2 (2.4)	3.2 (2.3)	3.2 (2.4)
Men	3.4 (2.4)	3.1 (2.5)	3.8 (2.5)	3.8 (2.5)	3.7 (2.4)	3.7 (2.4)
Women	2.5 (2.1)	2.1 (2.3)	2.7 (2.3)	2.7 (2.2)	2.7 (2.2)	2.7 (2.3)
At-risk alcohol drinkers, % (95% CI)						
Total sample	26.4 (25.0–27.9)	23.4 (22.1–24.8)	31.7 (29.6–33.9)	29.7 (27.6–31.8)	29.9 (28.5–31.3)	30.2 (28.8–31.6)
Men	26.7 (24.7–28.7)	24.2 (22.3–26.2)	31.7 (28.9–34.5)	29.2 (27.5–32.1)	29.3 (24.7–31.3)	29.2 (27.4–31.1)
Women	26.2 (24.2–28.3)	22.7 (20.9–24.7)	31.8 (28.7– 35.0)	30.1 (27.0–33.4)	30.5 (25.6–32.5)	31.1 (29.2–33.2)

AUDIT-C, Alcohol Use Disorder Identification Test; CI, confidence interval; SD, standard deviation.

^a^Survey 1: April–May 2020, t_−1_ corresponds to pre-lockdown (February–March, 2020) and t_0_ to the first lockdown period (March–May 2020); Survey 2: November–December 2020; Survey 3: May 2021; Survey 4: February–March 2022; Survey 5: April 2023.

^*^Unweighted counts for males and females are reported here, thus differing from the weighted ones presented in previous LOST in Italy studies.

When examining data by sex, similar patterns were observed for the prevalence of alcohol consumption and at-risk consumption. Men had consistently higher prevalence of alcohol use compared to women across all surveys. Conversely, the percentage of women with at-risk alcohol consumption was mainly comparable with that of men in all the time points, reaching even slightly higher values in the last surveys.

### Prospective cohort analysis

The figures obtained by analyzing the cohort of individuals participating in the first survey and in at least one of the following ones are mainly consistent with the overall prevalence estimates (eTable 2); however, the longitudinal sample deviates from representativeness criteria, and the results reported here do not allow for generalizability.

The associations between selected individual-level characteristics and being at-risk alcohol drinkers in the prospective cohort of 5,378 are presented in Table 2, overall and stratified by economic status. Compared to the pre-lockdown period (t_−1_), the lockdown survey (t_0_) showed a significant decrease in the odds of being at-risk alcohol drinkers (OR 0.77; 95% CI, 0.68–0.87). Significant increases were found at t_1_ (OR 1.51; 95% CI, 1.32–1.73), at t_2_ (OR 1.31; 95% CI, 1.13–1.50), at t_3_ (OR 1.27; 95% CI, 1.13–1.43), and at t_4_ (OR 1.27; 95% CI, 1.12–1.43). No significant difference was observed by sex in the total sample. However, stratifying by economic status, well-off men were statistically significantly less likely to be at-risk alcohol drinkers compared with well-off women (OR 0.66; 95% CI, 0.46–0.94). Being at-risk alcohol drinkers was inversely associated with the age group 35–54 years (OR 0.73; 95% CI, 0.60–0.89) compared to younger individuals. Being at-risk alcohol drinker was less frequent with decreasing economic status (*P* for trend <0.001), in subjects economically inactive (OR 0.83; 95% CI, 0.71–0.97) compared to those currently working, and among divorced/separated (OR 0.77; 95% CI, 0.60–0.99) and single (OR 0.75; 95% CI, 0.64–0.89), compared to married individuals. Within the well-off category, on the contrary, divorced/separated individuals were more likely to be at-risk drinkers (OR 2.25; 95% CI, 1.12–4.54). When considering mental distress indicators, individuals experiencing anxiety or depressive symptoms had significantly higher odds of being at-risk alcohol consumers (OR 1.24; 95% CI, 1.12–1.37), as well as those using psychotropic drugs (OR 1.99; 95% CI, 1.69–2.35). Similarly, the use of conventional cigarettes and novel products was associated with increased odds of being at-risk drinker when considering novel products (OR 1.55; 95% CI, 1.28–1.88), conventional cigarettes exclusively (OR 2.64; 95% CI, 2.29–3.05), and dual users (users of both conventional and novel products; OR 3.67; 95% CI, 3.00–4.48) compared to non-users. No significant differences emerged according to level of education and sleep disorders. Results stratified by sex, age class, and level of education are reported in eTable 3, eTable 4, and eTable 5. Compared to current female workers, retired women have significantly lower risk of alcohol disorders, while conversely, retired men are at a higher risk than workers, although not statistically significantly. Furthermore, divorced/separated women and single men are at a lower risk compared to their married counterparts (eTable 3). eTable 4 shows how a decreased risk of harmful alcohol use is statistically significantly associated with lower education levels only in the 35–54 age group. As reported in eTable 5, a lower risk was observed among individuals with low education who are single and among individuals with high education who are divorced/separated compared to their married counterparts. Additionally, the gradient of association with the use of psychotropic drugs decreased with decreasing education levels. Sensitivity analyses were conducted to assess the association of seasonality with risky alcohol consumption. Due to collinearity issues, the analysis was conducted by replacing the survey time variable with the season variable, which in our study assumed only two values: winter and spring. Results indicate lower odds of being risky alcohol drinkers in spring compared to winter (OR 0.86; 95% CI, 0.80–0.93), independently of the COVID-19 wave (eTable 6).

**Table 2.** Multilevel random intercept logistic regression for being at-risk alcohol drinker by survey period, socio-demographic and individual characteristics in the longitudinal cohort, total and by economic status, *N* = 5,378, Italy, 2020–2023

		Economic status			

	Total	Over the national mean	On average	Below the national mean	*P*
Period^a^		*N*(t_0_) = 742	*N*(t_0_) = 3,314	*N*(t_0_) = 1,322	
t_−1_ (pre-lockdown)	Ref.				
t_0_ (lockdown)	**0.77 (0.68–0.87)**	**0.66 (0.49–0.90)**	**0.81 (0.70–0.95)**	**0.69 (0.54–0.89)**	0.3917
t_1_	**1.51 (1.32–1.73)**	**1.59 (1.10–2.30)**	**1.59 (1.33–1.91)**	1.30 (0.97–1.74)	
t_2_	**1.31 (1.13–1.51)**	1.04 (0.71–1.52)	**1.51 (1.25–1.82)**	1.05 (0.77–1.43)	
t_3_	**1.27 (1.13–1.43)**	1.15 (0.83–1.58)	**1.32 (1.12–1.54)**	1.27 (0.98–1.65)	
t_4_	**1.27 (1.12–1.43)**	**1.43 (1.03–1.98)**	**1.23 (1.04–1.45)**	1.22 (0.93–1.60)	
Sex					
Women	Ref.				
Men	0.91 (0.78–1.06)	**0.66 (0.46–0.94)**	0.93 (0.77–1.12)	1.19 (0.87–1.61)	0.0273
Level of education					
High	Ref.				
Medium	0.89 (0.76–1.05)	0.97 (0.66–1.42)	0.96 (0.79–1.17)	0.83 (0.60–1.16)	0.7326
Low	0.87 (0.68–1.10)	1.27 (0.70–2.30)	0.94 (0.70–1.27)	0.70 (0.45–1.08)	
	*P* trend = 0.144		*P* trend = 0.648		
Age, years					
18–34	Ref.				
35–54	**0.73 (0.60–0.89)**	0.70 (0.46–1.06)	0.78 (0.62–1.00)	**0.53 (0.37–0.78)**	0.8798
55–74	0.87 (0.69–1.08)	0.77 (0.47–1.27)	0.86 (0.65–1.15)	0.68 (0.45–1.04)	
	*P* trend = 0.217				
Economic status					
Over the national mean	Ref.				
On average	**0.72 (0.62–0.84)**				
Below the national mean	**0.64 (0.53–0.77)**				
	*P* trend < 0.001				
Working condition					
Currently working	Ref.				
Economically inactive	**0.83 (0.71–0.97)**	0.86 (0.52–1.42)	**0.67 (0.53–0.85)**	0.99 (0.75–1.31)	0.4256
Retired	0.90 (0.72–1.12)	0.71 (0.39–1.29)	0.91 (0.68–1.21)	0.97 (0.61–1.53)	
Marital status					
Married	Ref.				
Divorced/separated	**0.77 (0.60–0.99)**	**2.25 (1.12–4.54)**	**0.62 (0.44–0.87)**	0.80 (0.52–1.24)	0.0190
Widowed	0.94 (0.51–1.73)	0.41 (0.08–2.19)	1.18 (0.55–2.53)	0.47 (0.15–1.52)	
Single	**0.75 (0.64–0.89)**	**0.51 (0.34–0.76)**	**0.70 (0.56–0.86)**	0.83 (0.60–1.14)	
Anxiety or depressive symptoms					
No	Ref.				
Yes	**1.24 (1.12–1.37)**	**1.51 (1.15–1.97)**	**1.28 (1.12–1.46)**	1.20 (0.97–1.48)	0.3715
Sleep disorders					
No	Ref.				
Yes	0.99 (0.90–1.09)	1.10 (0.85–1.42)	0.96 (0.84–1.08)	1.01 (0.82–1.24)	0.9233
Use of psychotropic drugs					
No	Ref.				
Yes	**1.99 (1.69–2.35)**	**4.05 (2.78–5.9)**	**2.82 (2.33–3.41)**	**2.88 (2.17–3.82)**	0.3540
Use of nicotine containing products					
Non-users	Ref.				
E-cigarette or HTP users	**1.55 (1.28–1.88)**	**1.77 (1.08–2.88)**	**1.74 (1.36–2.24)**	**1.62 (1.03–2.53)**	0.8384
Exclusive conventional tobacco smokers	**2.64 (2.29–3.05)**	**4.05 (2.78–5.90)**	**2.82 (2.33–3.41)**	**2.88 (2.17–3.82)**	
Dual users	**3.67 (3.00–4.48)**	**5.77 (3.45–9.65)**	**3.89 (2.98–5.10)**	**4.68 (3.03–7.22)**	

CI, confidence interval; HTP, heated tobacco products; OR, odds ratio; Ref., reference category.

^a^Survey 1: April–May 2020, t_−1_ corresponds to pre-lockdown (February–March, 2020) and t_0_ to the first lockdown period (March–May 2020); Survey 2: November–December 2020; Survey 3: May 2021; Survey 4: February–March 2022; Survey 5: April 2023.

OR and 95% CI were estimated from logistic random intercept model and adjusted by geographical area. Estimates in bold type are statistically significant at 0.05.

## DISCUSSION

Despite the abundance of studies conducted in several countries during the COVID-19 restriction periods,^33^ as far as we know no previous study investigated changes in behavior in the long-term after the pandemic. Our study showed a decline in alcohol use prevalence and at-risk alcohol drinking in Italy during the first and strictest lockdown and a peak increase in the following surveys, conducted throughout most of the COVID-19 pandemic waves, enabling us to capture the potential impact of pandemic peaks on population psychological distress and lifestyle changes. The prevalence observed in the last wave (between April 4 and May 3, 2023) are in line with other studies conducted on representative samples.^18^^,^^19^ Indeed, the greatest risk of at-risk drinking was observed in the period November–December 2020, when—after the start of the second pandemic wave and shortly before Christmas holidays—a number of psychologically and socially demanding regulations came together: the Italian government announced a new tightening of restrictions, the curfew was in force, the distance learning had become compulsory for secondary school, there was a ban on travel between regional territories, and the closure of shopping centers on weekends was ordered. The surveys carried out in the following 3 years—characterized by a relaxation of the pandemic-related limitations—showed that alcohol prevalence and at-risk consumption remained higher than that measured in the pre-lockdown period, particularly in women.

As COVID-19 restrictions gradually ease across Europe, according to the European Drug Report 2022,^34^ psychoactive substance consumption indicators generally point to a return to the pre-pandemic situation, after a modest reduction appeared to have accompanied COVID-19 restrictions. The same reduction was not measured in an equally generalized manner for legal substances, such as alcohol and tobacco,^29^ attributable to the constant availability of supply throughout the pandemic period. According to data published by the Organisation for Economic Co-operation and Development in 2021,^35^ selected demographic groups continued to exhibit certain patterns of harmful alcohol consumption; for instance, among women with higher education levels, as well as women situated at both ends of the income distribution spectrum. Lockdown measures have influenced both consumption patterns and sales of alcohol, with different effects in different geographic areas. While the Nielsen Company reported a 54% increase in alcohol sales in the United States in the first months of the restrictive measures, mainly due to online sales,^36^ government data from other countries suggest, on the one hand, an increase between 3% and 5% in alcohol sales. On the other hand, a reported decrease in the prevalence of binge drinking also emerged.^35^ A United States study has highlighted an increase in alcohol consumption among women, in the 30–59-years age group, and among non-Hispanic white subjects, between the pre-pandemic period (April–June 2019) and during the pandemic (May–June 2020). Among women, increases in excessive alcohol consumption were also detected, equivalent to an increase of one day for 1 in 5 women as well as in the Short Inventory of Problems score, indicative of an increase in alcohol-related problems regardless of the level of consumption, per almost 1 in 10 women.^37^ In Belgium, Vanderbruggen and colleagues found an increase in alcohol consumption during the lockdown inversely associated with increasing age and directly linked to a larger number of children in the home, unemployment, and work in non-healthcare sectors. A small but significant increase was also found in the number of drinks consumed per day during confinement.^38^ In France, the CoviPrev study found that, among alcohol consumers, between 10 and 16% had increased their consumption during the confinement period. Among those who increased consumption, who were more likely to be under 50 years old, resident in large urban areas, parents of children under 16 years old and with high levels of anxiety and depression, the majority declared that they had increased the frequency of drinking, rather than the number of consecutive drinks in 1 day.^39^^,^^40^ In the United Kingdom, a study identified risk factors associated with increased alcohol consumption during lockdown as being young, being female, having a high income, having anxiety disorders, and being worried about one’s finances.^41^

Although we found that the prevalence of drinkers was higher among men than among women, it is interesting to note that in men there was a difference of two percentage points more in the latest survey compared to before lockdown, while in women the difference was of four percentage points more. Similarly, the AUDIT-C test score in all surveys was lower among women than among men, but when looking at the percentage of individuals at-risk for harmful alcohol consumption, women figures exceed men ones in the long-term. Like what has been reported in previous studies, a significant association was found between being at-risk for alcohol disorders and female sex,^37^^,^^41^ as well as with the higher income category.^41^ Moreover, similar to previous studies, our results showed a lower risk of alcohol disorders with increasing age,^38^^,^^39^^,^^41^ decreasing income, and unemployment.^38^^,^^41^

Our results show a direct association between at-risk drinking and the increase in symptoms of anxiety and depression, and with the use of psychotropic drugs. The pandemic stress-related burden served as a comprehensive source of stress that differently affected individuals at personal, economic, and social level, highlighting individual fragilities and vulnerable groups.^8^^,^^12^^,^^20^^,^^42^^–^^44^ Alcohol use and misuse are well-known to be linked to increased levels of stress,^45^ and higher level of stress and anxiety probably contributed to the use of alcohol to cope with the pandemic situation.^46^ Similarly, increased levels of uncertainty caused by the 2008 financial crisis have led to an increase in the prevalence of binge drinking, despite an overall decrease in the prevalence of alcohol use, suggesting the possibility that more vulnerable segments of alcohol drinkers may increase their consumption of alcohol in a maladaptive direction.^47^^,^^48^

A study conducted on the first LOST survey linked worsening in smoking habits with mental distress.^29^ Therefore, it is not surprising the strong association between the risk for alcohol disorders and smoking behavior found in our study, with a gradient that increases among users of e-cigarettes or HTPs, exclusive smokers of conventional cigarettes and dual users, compared to the abstinent.

On the other hand, our results found a greater risk of hazardous alcohol consumption with being married than with any other marital status within the global cohort, even though the major risk was observed among well-off divorced/separated individuals. Although we are not aware of any indications to this effect in the literature, this result can be considered in line with studies that have found a similar association with features that could be considered proxies for being married, such as increasing number of children in the home,^38^ or with being parents of children under age 16 years.^39^

Many studies have linked alcoholism with seasonal abuse patterns^52^ and in relation to seasonal affective disorders,^53^ and a study by Sher (2002) highlighted a genetic basis linking alcohol dependence and seasonality.^54^ To address seasonal confounding factors, we conducted a sensitivity analysis by replacing the survey time variable with the season variable, which in our study assumed values winter or spring, corresponding to the main COVID-19 waves. The results indicate a link between at-risk alcohol consumption and the winter season even in the general population, regardless of the COVID-19 wave. A recent study by Gunnerlind and colleagues explored alcohol sales in three Nordic countries during the COVID-19 pandemic period, comparing them to the 2 years before the pandemic. While alcohol sales showed an initial increase at the beginning of the pandemic, comparison with previous years highlighted an overlap in seasonal sales patterns, with higher figures in winter and mid-summer, thus resulting in no relevant changes during COVID-19 period.^55^ Although our study does not allow for a comparison of risky alcohol consumption with previous years, we found an association with the winter season regardless of the survey time. This leads us to a more cautious interpretation of the relationship between survey time and risky alcohol consumption, considering the winter season, including Christmas time, as a confounding factor, without detracting from the potential effect of distress stemming from the health emergency period on specific vulnerable population subgroups, who appear to have been more susceptible to at-risk alcohol consumption like younger individuals, women with higher economic status, and married individuals.

### Strength and limitations

Among the limitations of the present study, the selection of the samples from online panels should be considered, since this could weaken the generalizability of the findings to the whole Italian adult population, despite the efforts implemented to obtain a representative sample. However, our results are in line with other national studies^49^ confirming the good degree of representativeness obtained in the LOST samples. Another limitation is represented by the fact that our analyses do not permit inferring causal relationships. Nonetheless, we were also able to conduct analyses on the cohort of individuals taking part to more than one survey, using a statistical model which allowed to account for the longitudinal structure of the data by considering the correlation within individuals. It should be highlighted that our results do not have a causal interpretation: changes in a variable would not lead to changes in the outcome of a magnitude represented by its regression coefficient. The interpretation of the model coefficients refer to an association between variables net to the each adjusting variable, thus avoiding the so-called table 2 fallacy problem (ie, when the causal effect of confounding factors is estimated in the model that, in our case, could be socio-demographic variables that may cause both changes in psychological and alcohol variables).^50^^,^^51^ Moreover, despite the limitation of having a reduced number of observations in the two middle COVID-19 waves, the modelling strategy allowed to include all the observations coming from surveys with different sample sizes in an unbalanced design, without loss of power, and with not equally spaced measurements. A further strength of the present study is represented by the fact that the surveys were carried out during most of the COVID-19 pandemic waves, allowing us to capture the possible effect of the pandemic peaks on the population psychological distress and lifestyle changes. Moreover, the study lies in the breadth of the samples and the use of validated scales for detecting several individual characteristics.

### Conclusion

Stress related to the COVID-19 pandemic has produced significant changes in alcohol use and misuse habits in the Italian adult population. While some studies have underlined an increase in the at-risk alcohol consumption among men in the short-term, the long-term results of our study indicate the need to pay particular attention also to women, young people, workers, individuals with higher income, married individuals, subjects with symptoms of anxiety and depression, as well as psychotropic substance and nicotine users. Understanding who experiences the lasting adverse effects of the COVID-19 pandemic and the conditions under which it occurs provides valuable insights into how effective psychological support can be implemented for individuals coping with traumatic events and the consequences of this and future social crises.

## SUPPLEMENTARY MATERIAL

eTable 1. Unweighted counts of individuals included in the sample according to sociodemographic characteristics, by survey, Italy, 2020–2023

eTable 2. Distribution of individuals included in the prospective cohort^b^ according to alcohol consumption and at-risk consumption, by sex and survey, Italy, 2020–2023

eTable 3. Multilevel random intercept logistic regression for being at-risk alcohol drinker by survey period, socio-demographic and individual characteristics in the longitudinal cohort, total and by sex, N = 5,378, Italy, 2020–2023

eTable 4. Multilevel random intercept logistic regression for being at-risk alcohol consumer by survey period, socio-demographic and individual characteristics in the longitudinal cohort, total and by age class, N = 5,378, Italy, 2020–2023

eTable 5. Multilevel random intercept logistic regression for being at-risk alcohol consumer by survey period, socio-demographic and individual characteristics in the longitudinal cohort, total and by level of education, N = 5,378, Italy, 2020–2023

eTable 6. Multilevel random intercept logistic regression for being at-risk alcohol drinker by season, socio-demographic and individual characteristics in the longitudinal cohort, N = 5,378, Italy, 2020–2023

eFigure 1. Correspondence between pandemic peaks and LOST surveys in Italy from 2020 to 2023
