# Supplementary material for: Changes in Alcohol Consumption During and After the Covid-19 Pandemic From 2020 to 2023 in a Prospective Cohort of Italian Adults
Source: J Epidemiol. 2025 Feb 5;35(2):63–70. doi: 10.2188/jea.JE20230340 (PMC11706678; doi:10.2188/jea.JE20230340)

eTable 1. Unweighted counts of individuals included in the sample according to socio-demographic characteristics, by survey, Italy, 2020–2023

	**Survey 1 t_{-1}[a]**	**Survey 1 t_0[a]**	**Survey 2 t_1[a]**	**Survey 3 t_2[a]**	**Survey 4 t_3[a]**	**Survey 5 t_4[a]**
Total sample, N	6,003	6,003	3,185	3,000	6,600	6,600
Men, N	3,026*	3,026	1,689	1,601	3,299	3,288
Women, N	2,977*	2,977	1,496	1,399	3,301	3,312
Level of education						
High	2,283	2,283	1,277	1,186	2,501	2,538
Medium	2,895	2,895	1,504	1,417	3,227	3,220
Low	825	825	404	397	872	842
Age, years						
18–34	1,456	1,456	757	679	1,537	1,508
35–54	2,760	2,760	1,497	1,405	3,110	3,134
55–74	1,787	1,787	931	916	1,953	1,958
Economic status						
Over the national mean	815	815	456	440	987	1,050
On average	3,699	3,699	1,962	1,855	4,054	3,999
Below the national mean	1,489	1,489	767	705	1,559	1,551
Working condition						
Currently working	4,166	4,166	2,247	2,149	4,779	4,818
Economically inactive	1,301	1,301	648	539	1,151	904
Retired	536	536	290	312	670	878
Marital Status						
Married	4,077	4,077	2,184	2,072	4,622	4,646
Divorced/separated	394	394	204	197	429	455
Widowed	82	82	43	42	96	110
Single	1,450	1,450	754	689	1,453	1,389

CI, confidence interval; SD, standard deviation.

[a] Survey 1: April-May 2020, t_{-1} corresponds to pre-lockdown (February–March, 2020) and t_0 to the first lockdown period (March–May 2020); Survey 2: November–December 2020; Survey 3: May 2021; Survey 4: February–March 2022; Survey 5: April 2023

*Unweighted counts for males and females are reported here, thus differing from the weighted ones presented in previous LOST in Italy studies.

eTable 2. Distribution of individuals included in the prospective cohort[b] according to alcohol consumption and at-risk consumption, by sex and survey, Italy, 2020–2023

	**Wave 1 t_{-1}[a]**	**Wave 1 t_0[a]**	**Wave 2 t_1[a]**	**Wave 3 t_2[a]**	**Wave 4 t_3[a]**	**Wave 5 t_4[a]**
Total sample, N	5,378	5,378	3,185	2,814	4,831	4,445
Men, N	2,747	2,747	1,689	1,518	2,487	2,299
Women, N	2,631	2,631	1,496	1,296	2,344	2,146
Alcohol drinkers, % (95% CI)						
Total sample	84.4 (83.4–85.3)	75.9 (74.7–77.0)	87.7 (86.5–88.8)	87.9 (86.7–89.1)	87.4 (86.5–88.3)	87.3 (86.3–88.2)
Men	90.5 (89.3–91.5)	83.7 (82.3–85.1)	93.3 (92.0–94.4)	92.0 (90.5–93.2)	92.1 (91.0–93.1)	92.5 (91.3–93.5)
Women	78.0 (76.5–79.6)	67.6 (65.8–69.4)	81.3 (79.2–83.2)	83.2 (81.0–85.1)	82.4 (80.8–83.9)	81.7 (80.0–83.3)
Score AUDIT-C, mean (SD)						
Total sample	2.9 (2.3)	2.7 (2.4)	3.3 (2.4)	3.2 (2.3)	3.2 (2.3)	3.2 (2.4)
Men	3.4 (2.3)	3.1 (2.5)	3.8 (2.5)	3.7 (2.4)	3.6 (2.3)	3.7 (2.4)
Women	2.5 (2.1)	2.1 (2.3)	2.7 (2.2)	2.6 (2.2)	2.7 (2.2)	2.7 (2.3)
At-risk alcohol drinkers, % (95% CI)						
Total sample	25.5 (24.3–26.7)	23.4 (22.2–24.5)	30.3 (28.8–31.9)	28.7 (27.1–30.4)	28.8 (27.5–30.1)	29.1 (27.7–30.4)
Men	25.3 (23.7–27.0)	23.7 (22.2–25.4)	30.7 (28.5–32.9)	28.6 (26.4–30.9)	27.0 (25.3–28.8)	28.2 (26.4–30.1)
Women	25.7 (24,1–27.4)	23.0 (21.4–24.7)	29.9 (27.7–32.3)	28.8 (26.5–31.4)	30.6 (28.8–32.5)	30.0 (28.1–31.9)

CI, confidence interval; SD, standard deviation.

[a] Survey 1: April–May 2020, t_{-1} corresponds to pre-lockdown (February–March, 2020) and t_0 to the first lockdown period (March–May 2020); Survey 2: November–December 2020; Survey 3: May 2021; Survey 4: February–March 2022; Survey 5: April 2023

[b] Individuals participating in Survey 1 and at least another one of the following surveys

eTable 3. Multilevel random intercept logistic regression for being at-risk alcohol drinker by survey period, socio-demographic and individual characteristics in the longitudinal cohort, total and by sex, N=5,378, Italy, 2020–2023

	**Total**	**Sex**	
		**Women**	**Men**
Period[a]		N(t_0) = 2,977	N(t_0) = 3,026
t_{-1} (pre-lockdown)	Ref.	Ref.	Ref.
t_0 (lockdown)	**0.77 (0.68–0.87)**	**0.74 (0.62–0.89)**	**0.80 (0.68–0.94)**
t_1	**1.51 (1.32–1.73)**	**1.52 (1.24–1.86)**	**1.50 (1.25–1.80)**
t_2	**1.31 (1.13–1.51)**	**1.34 (1.08–1.66)**	**1.27 (1.05–1.54)**
t_3	**1.27 (1.13–1.43)**	**1.50 (1.26–1.78)**	1.10 (0.93–1.30)
t_4	**1.27 (1.12–1.43)**	**1.36 (1.14–1.64)**	**1.19 (1.01–1.41)**
Sex			
Women	Ref.		
Men	0.91 (0.78–1.06)		
Level of education			
High	Ref.	Ref.	Ref.
Medium	0.89 (0.76–1.05)	0.84 (0.65–1.08)	0.90 (0.73–1.12)
Low	0.87 (0.68–1.10)	0.73 (0.51–1.06)	0.96 (0.71–1.29)
Age, years			
18–34	Ref.	Ref.	Ref.
35–54	**0.73 (0.60–0.89)**	**0.69 (0.53–0.90)**	0.77 (0.59–1.02)
55–74	0.87 (0.69–1.08)	0.86 (0.61–1.22)	0.89 (0.66–1.20)
Economic status			
Over the national mean	Ref.	Ref.	Ref.
On average	**0.72 (0.62–0.84)**	**0.58 (0.46–0.74)**	**0.82 (0.68–0.99)**
Below the national mean	**0.64 (0.53–0.77)**	**0.50 (0.38–0.67)**	**0.77 (0.60–0.98)**
Working condition			
Currently working	Ref.	Ref.	Ref.
Economically inactive	**0.83 (0.71–0.97)**	0.86 (0.70–1.06)	0.78 (0.60–1.02)
Retired	0.90 (0.72–1.12)	**0.66 (0.46–0.93)**	1.06 (0.80–1.41)
Marital Status			
Married	Ref.	Ref.	Ref.
Divorced/separated	**0.77 (0.60–0.99)**	**0.71 (0.50–0.99)**	0.86 (0.60–1.22)
Widowed	0.94 (0.51–1.73)	1.12 (0.57–2.22)	1.19 (0.41–3.47)
Single	**0.75 (0.64–0.89)**	0.85 (0.67–1.08)	**0.69 (0.55–0.86)**
Anxiety or depressive symptoms			
No	Ref.	Ref.	Ref.
Yes	**1.24 (1.12–1.37)**	**1.20 (1.04–1.38)**	**1.30 (1.13–1.50)**
Sleep Disorders			
No	Ref.	Ref.	Ref.
Yes	0.99 (0.90–1.09)	0.92 (0.80–1.07)	1.08 (0.94–1.22)
Use of Psychotropic Drugs			
No	Ref.	Ref.	Ref.
Yes	**1.99 (1.69–2.35)**	**1.90 (1.47–2.46)**	**2.08 (1.66–2.61)**
Use of nicotine containing products			
Non-users	Ref.	Ref.	Ref.
E-cigarettes or HTP users	**1.55 (1.28–1.88)**	**1.55 (1.15–2.10)**	**1.56 (1.21–2.01)**
Exclusive conventional tobacco smokers	**2.64 (2.29–3.05)**	**3.04 (2.46–3.74)**	**2.37 (1.95–2.88)**
Dual users	**3.67 (3.00–4.48)**	**4.28 (3.18–5.77)**	**3.34 (2.56–4.37)**

CI, confidence interval; HTP, heated tobacco products; OR, odds ratio; Ref., reference category.

[a] Survey 1: April–May 2020, t_{-1} corresponds to pre-lockdown (February–March, 2020) and t_0 to the first lockdown period (March–May 2020); Survey 2: November–December 2020; Survey 3: May 2021; Survey 4: February–March 2022; Survey 5: April 2023

OR and 95% CI were estimated from logistic random intercept model and adjusted by geographical area. Estimates in bold type are statistically significant at 0.05

eTable 4. Multilevel random intercept logistic regression for being at-risk alcohol consumer by survey period, socio-demographic and individual characteristics in the longitudinal cohort, total and by age class, N=5,378, Italy, 2020–2023

	**Total**	**Age class, years**		
		**18–34**	**35–54**	**55–74**
Period[a]		$N(t_0)$ = 1,239	$N(t_0)$ = 2,478	$N(t_0)$ = 1,661
t_{-1} (pre-lockdown)	Ref.	Ref.	Ref.	Ref.
t_0 (lockdown)	**0.77 (0.68–0.87)**	**0.46 (0.36–0.59)**	0.93 (0.78–1.11)	0.89 (0.71–1.10)
t_1	**1.51 (1.32–1.73)**	1.11 (0.84–1.46)	**1.60 (1.31–1.95)**	**1.81 (1.41–2.34)**
t_2	**1.31 (1.13–1.51)**	1.10 (0.82–1.47)	**1.40 (1.13–1.72)**	**1.36 (1.04–1.76)**
t_3	**1.27 (1.13–1.43)**	1.10 (0.86–1.40)	**1.43 (1.20–1.70)**	1.23 (0.99–1.53)
t_4	**1.27 (1.12–1.43)**	1.16 (0.90–1.50)	**1.44 (1.20–1.73)**	1.16 (0.92–1.46)
Sex				
Women	Ref.	Ref.	Ref.	Ref.
Men	0.91 (0.78–1.06)	0.76 (0.56–1.01)	0.92 (0.74–1.15)	1.01 (0.73–1.38)
Level of education				
High	Ref.	Ref.	Ref.	Ref.
Medium	0.89 (0.76–1.05)	0.78 (0.58–1.06)	**0.78 (0.61–0.98)**	1.26 (0.91–1.73)
Low	0.87 (0.68–1.10)	1.22 (0.75–1.99)	**0.57 (0.41–0.81)**	1.27 (0.79–2.05)
Age, years				
18–34	Ref.			
35–54	**0.73 (0.60–0.89)**			
55–74	0.87 (0.69–1.08)			
Economic status				
Over the national mean	Ref.	Ref.	Ref.	Ref.
On average	**0.72 (0.62–0.84)**	**0.70 (0.53–0.93)**	**0.72 (0.58–0.90)**	**0.71 (0.53–0.94)**
Below the national mean	**0.64 (0.53–0.77)**	**0.68 (0.47–0.96)**	**0.65 (0.49–0.87)**	**0.56 (0.39–0.80)**
Working condition				
Currently working	Ref.	Ref.	Ref.	Ref.
Economically inactive	**0.83 (0.71–0.97)**	0.86 (0.65–1.12)	**0.76 (0.60–0.98)**	1.05 (0.75–1.47)
Retired	0.90 (0.72–1.12)	1.66 (0.39–7.14)	0.96 (0.42–2.18)	0.95 (0.73–1.24)
Marital Status				
Married	Ref.			
Divorced/separated	**0.77 (0.60–0.99)**	1.07 (0.50–2.31)	**0.56 (0.38–0.84)**	0.97 (0.66–1.41)
Widowed	0.94 (0.51–1.73)	2.43 (0.43–13.85)	**0.30 (0.10–0.90)**	1.29 (0.64–2.61)
Single	**0.75 (0.64–0.89)**	0.91 (0.72–1.16)	**0.66 (0.51–0.85)**	0.73 (0.49–1.09)
Anxiety or depressive symptoms				
No	Ref.	Ref.	Ref.	Ref.
Yes	**1.24 (1.12–1.37)**	1.20 (0.99–1.46)	**1.27 (1.10–1.47)**	**1.23 (1.01–1.50)**
Sleep Disorders				
No	Ref.	Ref.	Ref.	Ref.
Yes	0.99 (0.90–1.09)	0.91 (0.75–1.10)	0.95 (0.82–1.09)	1.08 (0.90–1.30)
Use of Psychotropic Drugs				
No	Ref.	Ref.	Ref.	Ref.
Yes	**1.99 (1.69–2.35)**	**3.67 (2.64–5.12)**	**1.79 (1.39–2.32)**	**1.37 (1.01–1.86)**
Use of nicotine containing products				
Non-users	Ref.	Ref.	Ref.	Ref.
E-cigarettes or HTP users	**1.55 (1.28–1.88)**	**2.24 (1.58–3.17)**	**1.70 (1.29–2.24)**	0.92 (0.6–1.42)
Exclusive conventional tobacco smokers	**2.64 (2.29–3.05)**	**3.59 (2.73–4.73)**	**2.88 (2.33–3.56)**	**1.86 (1.39–2.47)**
Dual users	**3.67 (3.00–4.48)**	**5.22 (3.64–7.49)**	**3.85 (2.86–5.18)**	**2.67 (1.76–4.05)**

CI, confidence interval; HTP, heated tobacco products; OR, odds ratio; Ref., reference category.

[a] Survey 1: April-May 2020, t_{-1} corresponds to pre-lockdown (February–March, 2020) and t_0 to the first lockdown period (March–May 2020); Survey 2: November–December 2020; Survey 3: May 2021; Survey 4: February–March 2022; Survey 5: April 2023. OR and 95% CI were estimated from logistic random intercept model and adjusted by geographical area. Estimates in bold type are statistically significant at 0.05

eTable 5. Multilevel random intercept logistic regression for being at-risk alcohol consumer by survey period, socio-demographic and individual characteristics in the longitudinal cohort, total and by level of education, N=5,378, Italy, 2020–2023

	Total	Level of education		
		**Low**	**Medium**	**High**
Period[a]		N(t_0) = 2,069	N(t_0) = 2,574	N(t_0) = 735
t_{-1} (pre-lockdown)	Ref.	Ref.	Ref.	Ref.
t_0 (lockdown)	**0.77 (0.68–0.87)**	**0.80 (0.66–0.97)**	**0.76 (0.64–0.91)**	**0.72 (0.52–0.99)**
t_1	**1.51 (1.32–1.73)**	**1.55 (1.25–1.92)**	**1.64 (1.35–2.00)**	1.07 (0.73–1.57)
t_2	**1.31 (1.13–1.51)**	**1.54 (1.23–1.93)**	**1.29 (1.05–1.59)**	0.79 (0.53–1.18)
t_3	**1.27 (1.13–1.43)**	**1.39 (1.15–1.68)**	**1.24 (1.04–1.48)**	1.08 (0.78–1.50)
t_4	**1.27 (1.12–1.43)**	**1.43 (1.18–1.74)**	**1.26 (1.05–1.50)**	0.93 (0.66–1.32)
Sex				
Women	Ref.	Ref.	Ref.	Ref.
Men	0.91 (0.78–1.06)	0.89 (0.71–1.12)	0.88 (0.70–1.11)	1.2 (0.76–1.89)
Level of education				
High	Ref.			
Medium	0.89 (0.76–1.05)			
Low	0.87 (0.68–1.10)			
Age, years				
18–34	Ref.	Ref.	Ref.	Ref.
35–54	**0.73 (0.60–0.89)**	0.84 (0.63–1.11)	0.79 (0.59–1.06)	**0.38 (0.21–0.70)**
55–74	0.87 (0.69–1.08)	**0.59 (0.41–0.84)**	1.14 (0.83–1.57)	0.93 (0.45–1.91)
Economic status				
Over the national mean	Ref.	Ref.	Ref.	Ref.
On average	**0.72 (0.62–0.84)**	**0.70 (0.57–0.87)**	**0.79 (0.62–1.00)**	**0.56 (0.34–0.92)**
Below the national mean	**0.64 (0.53–0.77)**	**0.69 (0.51–0.94)**	**0.68 (0.52–0.90)**	**0.49 (0.28–0.84)**
Working condition				
Currently working	Ref.	Ref.	Ref.	Ref.
Economically inactive	**0.83 (0.71–0.97)**	0.77 (0.57–1.05)	0.87 (0.70–1.08)	0.82 (0.55–1.25)
Retired	0.90 (0.72–1.12)	0.98 (0.66–1.47)	0.88 (0.64–1.23)	0.71 (0.41–1.22)
Marital Status				
Married	Ref.	Ref.	Ref.	Ref.
Divorced/separated	**0.77 (0.60–0.99)**	0.82 (0.50–1.33)	0.87 (0.63–1.20)	**0.43 (0.22–0.83)**
Widowed	0.94 (0.51–1.73)	0.37 (0.09–1.45)	0.85 (0.36–1.99)	1.27 (0.47–3.43)
Single	**0.75 (0.64–0.89)**	**0.68 (0.54–0.87)**	0.84 (0.66–1.07)	0.81 (0.48–1.37)
Anxiety or depressive symptoms				
No	Ref.	Ref.	Ref.	Ref.
Yes	**1.24 (1.12–1.37)**	**1.24 (1.06–1.45)**	**1.17 (1.01–1.36)**	**1.47 (1.10–1.96)**
Sleep Disorders				
No	Ref.	Ref.	Ref.	Ref.
Yes	0.99 (0.90–1.09)	0.97 (0.84–1.13)	1.03 (0.89–1.19)	0.95 (0.72–1.24)
Use of Psychotropic Drugs				
No	Ref.	Ref.	Ref.	Ref.
Yes	**1.99 (1.69–2.35)**	**2.32 (1.8–3.00)**	**1.97 (1.53–2.55)**	1.41 (0.87–2.29)
Use of nicotine containing products				
Non-users	Ref.	Ref.	Ref.	Ref.
E-cigarettes or HTP users	**1.55 (1.28–1.88)**	**1.41 (1.06–1.87)**	**1.67 (1.25–2.24)**	**2.40 (1.31–4.39)**
Exclusive conventional tobacco smokers	**2.64 (2.29–3.05)**	**3.05 (2.43–3.83)**	**2.52 (2.04–3.10)**	**2.16 (1.45–3.21)**
Dual users	**3.67 (3.00–4.48)**	**5.01 (3.62–6.94)**	**2.84 (2.14–3.76)**	**3.96 (2.25–6.97)**

CI, confidence interval; HTP, heated tobacco products; OR, odds ratio; Ref., reference category.

[a] Survey 1: April–May 2020, t_{-1} corresponds to pre-lockdown (February–March, 2020) and t_0 to the first lockdown period (March–May 2020); Survey 2: November–December 2020; Survey 3: May 2021; Survey 4: February–March 2022; Survey 5: April 2023. OR and 95% CI were estimated from logistic random intercept model and adjusted by geographical area. Estimates in bold type are statistically significant at 0.05

eTable 6. Multilevel random intercept logistic regression for being at-risk alcohol drinker by season, socio-demographic and individual characteristics in the longitudinal cohort, N=5,378, Italy, 2020–2023

	**OR (95% CI)**
Season	
Winter	Ref.
Spring	**0.86 (0.80–0.93)**
Sex	
Women	Ref.
Men	0.91 (0.78–1.06)
Level of education	
High	Ref.
Medium	0.89 (0.76–1.05)
Low	0.86 (0.68–1.09)
Age, years	
18–34	Ref.
35–54	**0.73 (0.60–0.89)**
55–74	**0.83 (0.67–1.04)**
Economic status	
Over the national mean	Ref.
On average	**0.71 (0.61–0.82)**
Below the national mean	**0.64 (0.53–0.76)**
Working condition	
Currently working	Ref.
Economically inactive	**0.79 (0.68–0.93)**
Retired	0.96 (0.77–1.20)
Marital Status	
Married	Ref.
Divorced/separated	0.79 (0.61–1.01)
Widowed	0.89 (0.49–1.64)
Single	**0.74 (0.63–0.87)**
Anxiety or depressive symptoms	
No	Ref.
Yes	**1.19 (1.08–1.31)**
Sleep Disorders	
No	Ref.
Yes	1.03 (0.94–1.13)
Use of Psychotropic Drugs	
No	Ref.
Yes	**1.96 (1.66–2.31)**
Use of nicotine containing products	
Non-users	Ref.
E-cigarettes or HTP users	**1.41 (1.16–1.71)**
Exclusive conventional tobacco smokers	**2.54 (2.20–2.93)**
Dual users	**4.22 (3.46–5.14)**

CI, confidence interval; HTP, heated tobacco products; OR, odds ratio; Ref., reference category.
OR and 95% CI were estimated from logistic random intercept model and adjusted by geographical area.
Estimates in bold type are statistically significant at 0.05

eFigure 1. Correspondence between pandemic peaks and LOST surveys in Italy from 2020 to 2023

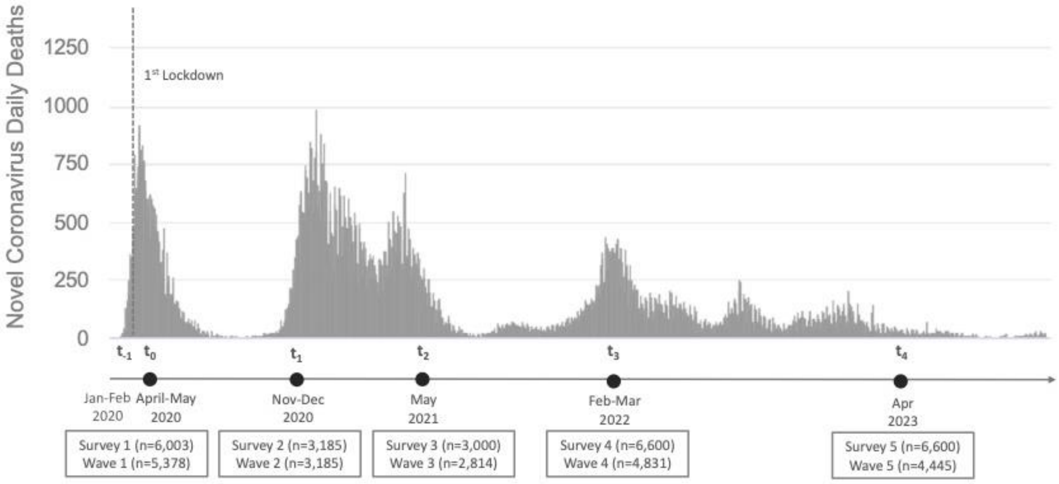

Supplement: Supplementary file 1 [file je-35-063-s001.pdf]
